# Canine edge width and height affect dental esthetics in maxillary canine substitution treatment

**DOI:** 10.1186/s40510-019-0268-y

**Published:** 2019-04-08

**Authors:** Ruomei Li, Li Mei, Pengfei Wang, Jiarong He, Qingyan Meng, Linna Zhong, Wei Zheng, Yu Li

**Affiliations:** 1 State Key Laboratory of Oral Diseases, National Clinical Research Center for Oral Diseases, Department of Orthodontics, West China Hospital of Stomatology, Sichuan University, Chengdu, China; 2 Discipline of Orthodontics, Department of Oral Sciences, Sir John Walsh Research Institute, Faculty of Dentistry, University of Otago, Dunedin, New Zealand; 3 State Key Laboratory of Oral Diseases, National Clinical Research Center for Oral Diseases, Department of Oral and Maxillofacial Surgery, West China Hospital of Stomatology, Sichuan University, No.14, South Renmin Road section 3, Chengdu, 610041 China; 4 Department of Orthodontics, Ninth People’s Hospital, School of Stomatology, Shanghai key Laboratory of Stomatology, Shanghai Jiao Tong University, Shanghai, China

## Abstract

**Background:**

To investigate the effect of canine edge width and height on dental esthetics in maxillary canine substitution treatment.

**Methods:**

A total of 127 canine substitution treatment cases were screened and evaluated by a panel of orthodontic experts and laypersons in the pilot study. The top five subjects with the esthetically most pleasant canine substitution were included in the study, resulting in 140 computerized images displaying only the upper dentition, with different canine edge widths (0%, 12.5%, 25%, 37.5%, 50%, 62.5%, and 75% of the central clinical width) and heights (− 0.5 mm, 0 mm, 0.5 mm, and 1.0 mm vertically relative to the central incisor edge) finally used for the esthetic evaluation by 101 observers (41 orthodontists and 60 laypersons). The ordered logistic regression analysis, the univariate analysis of variance, the chi-square, and Fisher’s exact tests were used for statistical analyses.

**Results:**

The most esthetic canine shape for canine substitution was found to be a shape with the edge width of 62.5% of the central incisor width and the edge height of 0.5 mm gingival to the central incisor edge (*P* < 0.05). The canine edge width of 50–75% and height of 0.5–0 mm gingival to the central incisor edge were generally considered to be esthetic by all observers. Orthodontists and laypersons had the same ranking on the top two most esthetic canine shapes (edge width and height 62.5% and 0.5 mm gingival; 50% and 0 mm incisal) as well as the bottom two most unesthetic canine shape (0% and 0.5 mm gingival; 75% and 1 mm incisal). Male and female observers generally had similar esthetic grades and rankings on the canine shapes (*P* > 0.05).

**Conclusions:**

The most esthetic canine shape for canine substitution is a shape with the canine edge width of 62.5% of the central incisor width and the edge height of 0.5 mm gingival to the central incisor edge. The different collocations of the canine edge width and height affect dental esthetics of the canine during canine substitution treatment.

## Background

The maxillary front teeth play an essential role in dentofacial esthetics [1]. For example, the absence of one or both maxillary lateral incisors (either congenitally or as a result of being extracted for reasons such as diminutive tooth) can significantly impact the dentofacial appearance, oral function, psychological well-being, and quality of life of the patient [2–5]. The treatments for these missing maxillary lateral incisors usually include prosthetic replacements (e.g., implantation, fixed, or removable dentures) or orthodontic space closure with canine substitution [6–10].

Numerous studies have demonstrated the advantages of canine substitution to replace the missing maxillary lateral incisor, including good esthetics [11, 12], long-term periodontal and temporomandibular joint health [13–15], and the avoidance of prosthetic materials in the oral cavity after treatment [16, 17], as well as reduced costs in treatment [16].

A challenge of canine substitution in practice is to recontour the shape of the canine after space closure by grinding and reshaping the canine enamel to produce esthetically pleasant edge width and height [10, 18, 19]. Clinicians usually use the maxillary central incisors as the reference for recontouring after the lateralization of the canines [10, 18]. However, it is still unclear what canine edge width and height is the most esthetic shape to the patients as well as to the orthodontists. A number of previous studies have found that the shade, gingival height, symmetry, crown width, crown height, and tip morphology of the substituted canine could affect the esthetic treatment outcome [19–23]. And there was a discrepancy in the esthetic perception between laypersons and orthodontists [24]. The laypersons were found to be not as perceptive and critical as the orthodontists when evaluating smile esthetics [11]. A result that was considered by orthodontists to be less than ideal may well be perfectly acceptable to the layperson [11, 22, 24]. It is important to understand the influence of the canine shape on dental esthetics during the patient-centered treatment of canine substitution [25, 26].

The aim of the study was to investigate the influence of the canine edge width and height on dental esthetics in canine substitution treatment.

## Material and methods

### Image preparation

The study was approved by the Ethics Committee of the State Key Laboratory of Oral Disease, West China School of Stomatology, Sichuan University, China. A total of 127 orthodontic patients from the Department of Orthodontics, West China Hospital of Stomatology, Sichuan University, were screened for eligibility. The inclusion criteria are as follows: (1) subjects must have full permanent dentition with two congenitally missing maxillary lateral incisors, (2) fixed orthodontic treatment involving canine substitution and shape modification to replace the missing lateral incisors, and (3) intraoral photographs were taken after the treatment and were of good quality. The exclusion criteria are as follows: (1) craniofacial defects or syndromes and (2) a history of restorative treatment on the anterior teeth.

**Fig. 1:**
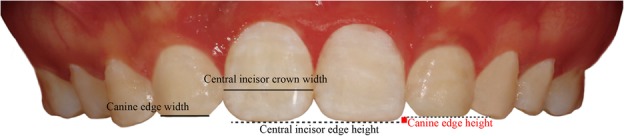
The esthetically most pleasant (ranked as top 1) canine shape with the highest grades for the incisal width and height by all observers in the study. The canine edge width was based on the percentage of the width of the maxillary central incisor clinical crown; the canine edge height was defined as the vertical distance of the incisal edges between the maxillary canine and central incisor

**Fig. 2:**
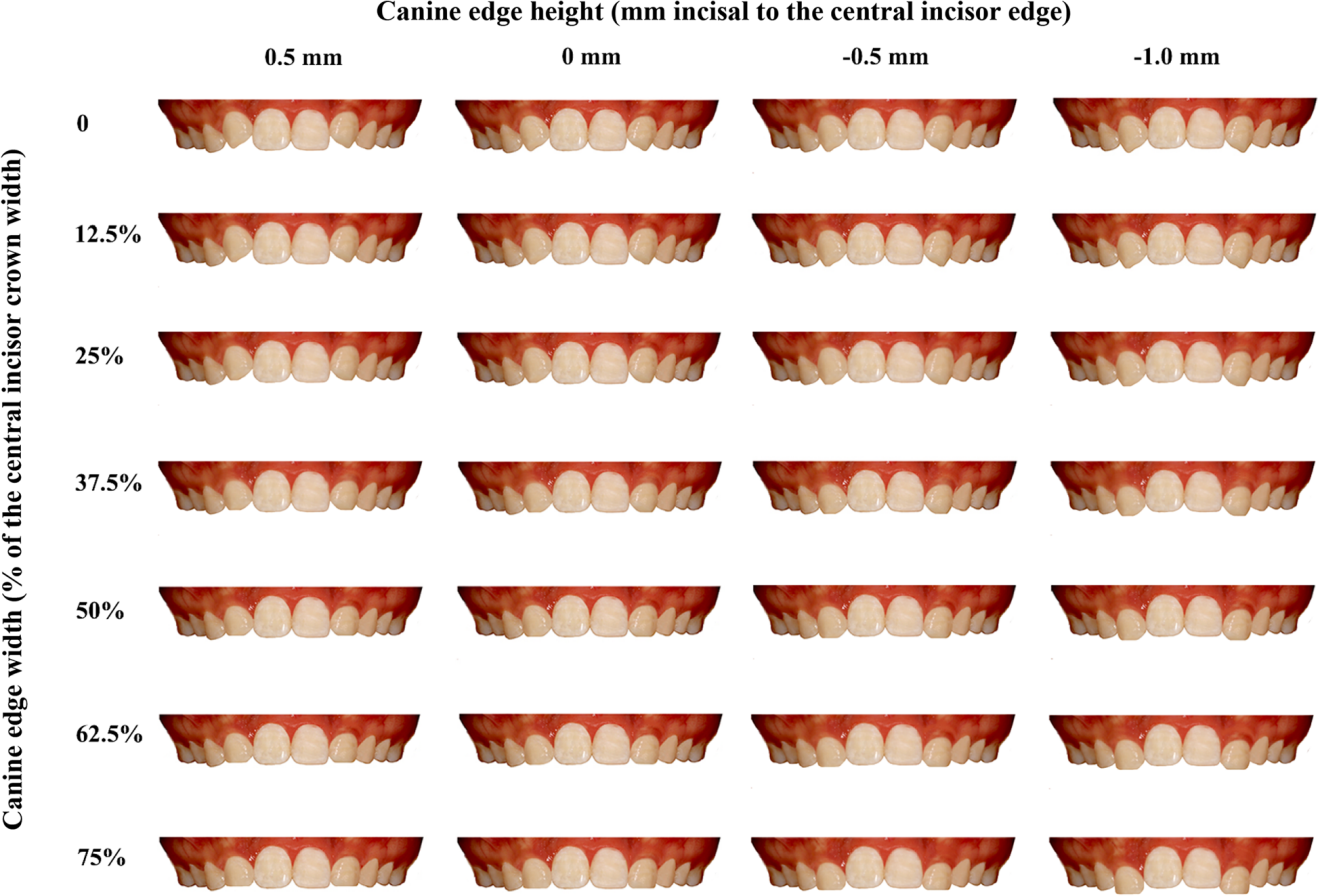
Example of the images used in the study. The canine edge width was based on the percentage of the incisal width of the maxillary central incisor and categorized to 0%, 12.5%, 25%, 37.5%, 50%, 62.5%, and 75% of the central incisor width. The canine edge height was defined as the distance of the incisal edges between the maxillary canine and central incisor, and categorized to − 0.5 mm, 0 mm, 0.5 mm, and 1.0 mm

The posttreatment intraoral frontal photo of each subject was cut to display only the upper dentition using Adobe Photoshop CS6 (Adobe Systems Inc. San Jose, CA, USA) (Fig. 1). These images were then evaluated by a panel of orthodontic experts and laypersons in the pilot study, and the top five subjects with the esthetically most pleasant canine substitution were included in the study. The images of those patients were subsequently processed using a computer to generate a series of images with different incisal edge width and height of the canine (Fig. 2).

The canine edge width was based on the percentage of the width of the maxillary central incisor clinical crown and categorized to 0%, 12.5%, 25%, 37.5%, 50%, 62.5%, and 75% of the central incisor width. The canine edge height was defined as the vertical distance of the incisal edges between the maxillary canine and central incisor, and categorized to − 0.5 mm (0.5 mm gingival to the central incisor edge), 0 mm (the same height with the central incisor edge), 0.5 mm (0.5 mm incisal to the central incisor edge), and 1.0 mm (1.0 mm incisal to the central incisor edge) (Figs. 1 and 2).

A total of 140 images (28 for each subject) were finally prepared and included for the esthetic evaluation and analyses.

### Participants and evaluation procedures

A total of 101 participants, including 41 orthodontists (20 male and 21 female, age range 25–30 years) and 60 laypersons (30 male and 30 female, age range 20–30 years) with similar socioeconomic status, were recruited to assess and grade these images based on the dental esthetics.

Each observer was asked to view and score the images using PowerPoint slides (with one image on each slide) in the same face-to-face setting. A blank slide was displayed between each image to minimize the disturbance from the former to the latter. These observers firstly viewed 10 random slides (3 s for each) to become familiar with the images before formally viewing and grading (5, very attractive; 4, attractive; 3, fair; 2, poor; 1, very poor) all the images (5 s for each) as previously described in the literature [27]. The formal assessment was subsequently repeated to allow the observers to check and modify their grades. The entire evaluation process was repeated again in the same setting 2 weeks later. The consistency of the grades was excellent for the orthodontists (Cronbach’s *α* = 0.95) and was good for the laypersons (Cronbach’s *α* = 0.85).

### Statistical analysis

Stata 14.0 (Stata Corp, Texas, TX, USA) and SPSS 21.0 (Statistical Package for the Social Sciences, SPSS Inc., Chicago, IL, USA) were used for the statistical analysis of the data. Ordered logistic regression analysis and the univariate analysis of variance were performed to analyze the effect of canine edge width and height on dental esthetics. Subgroup analyses were performed based on the observer’s background (orthodontist v.s. layperson) and gender (male v.s. female) by using the chi-square and Fisher’s exact tests. *P* values of less than 0.05 were considered statistically significant.

## Results

### Canine edge width and height

The canine edge widths of 50% and 62.5% of the central incisor width were ranked as top 1 and 2, respectively, by all observers (Table 1 and Fig. 2). The canine edge heights of 0 mm and − 0.5 mm incisal to the central incisor edge were ranked as top 1 and 2, respectively, by all observers (Table 1 and Fig. 2).

**Table 1:** Esthetic grade (mean ± standard deviation) and rank on the independent canine shape parameters (edge width and height) by orthodontists and laypersons

Canine shape parameters	All observers		Orthodontist		Layperson		Male		Female	
	Grade	Rank	Grade	Rank	Grade*	Rank	Grade*	Rank	Grade*	Rank
Edge width										
0%	2.92 ± 1.09	6	2.98 ± 0.91	6	2.84 ± 1.21	5	2.86 ± 1.17	6	2.93 ± 1.03	6
12.5%	3.06 ± 0.99	5	3.20 ± 1.78	5	2.79 ± 1.10	6	2.87 ± 1.00	5	3.04 ± 0.96	5
25%	3.14 ± 1.04	4	3.27 ± 0.83	4	2.91 ± 1.16	4	2.94 ± 1.10	4	3.18 ± 0.97	4
37.5%	3.21 ± 1.11	3	3.36 ± 1.04	3	2.98 ± 1.13	3	3.05 ± 1.08	3	3.23 ± 1.12	3
50%	3.43 ± 1.18	1	3.53 ± 1.39	1	3.36 ± 0.98	1	3.40 ± 1.05	1	3.46 ± 1.27	1
62.5%	3.30 ± 1.29	2	3.37 ± 1.37	2	3.26 ± 1.23	2	3.22 ± 1.18	2	3.38 ± 1.36	2
75%	2.74 ± 1.34	7	2.77 ± 1.48	7	2.72 ± 1.23	7	2.72 ± 1.23	7	2.76 ± 1.41	7
Edge height										
− 0.5 mm	3.15 ± 1.25	2	3.40 ± 1.24	2	3.02 ± 1.22	2	3.08 ± 1.23	2	3.25 ± 1.25	2
0 mm	3.46 ± 1.03	1	3.86 ± 0.88	1	3.19 ± 1.06	1	3.31 ± 1.04	1	3.61 ± 1.00	1
0.5 mm	3.13 ± 1.03	3	3.30 ± 0.87	3	2.99 ± 1.13	3	3.05 ± 1.08	3	3.18 ± 0.99	3
1.0 mm	2.55 ± 1.16	4	2.29 ± 0.99	4	2.73 ± 1.22	4	2.58 ± 1.10	4	2.52 ± 1.19	4

*Data represent mean ± standard deviation

The ordered logistic regression analysis revealed that both the canine edge width (odds ratio = 1.21, 95% confidence interval (95% CI) = 1.19–1.23, *P* = 0.003) and canine edge height (odds ratio = 1.93, 95% CI = 1.84–2.02, *P* = 0.001) had statistically significant influence on the dental esthetics. The univariate analysis of variance showed that the different collocations of these two canine shape parameters (i.e., edge width and height) also influenced the dental esthetics (Table 2).

**Table 2:** Esthetic grade (mean ± standard deviation) and rank on the canine shape by all observers

Canine shape		All observers	
Edge width (%)	Edge height (mm)	Grade	Rank
62.5	− 0.5	4.38 ± 0.60	1
50	0	4.17 ± 0.76	2
50	− 0.5	4.08 ± 0.75	3
62.5	0	3.89 ± 0.48	4
75	− 0.5	3.88 ± 0.64	5
37.5	0	3.76 ± 0.69	6
25	0	3.64 ± 0.27	7
0	0.5	3.47 ± 0.24	8
75	0	3.45 ± 0.46	9
37.5	0.5	3.40 ± 0.70	10
12.5	0.5	3.38 ± 0.23	11
25	0.5	3.27 ± 0.52	12
12.5	1.0	3.23 ± 0.22	13
0	1.0	3.22 ± 0.19	14
50	0.5	3.22 ± 0.26	15
37.5	− 0.5	3.02 ± 0.11	16
62.5	0.5	2.92 ± 0.33	17
12.50	0	2.92 ± 0.19	18
0	0	2.92 ± 0.30	19
25	1.0	2.72 ± 0.25	20
25	− 0.5	2.66 ± 0.30	21
37.5	1.0	2.44 ± 0.34	22
12.50	− 0.5	2.43 ± 0.11	23
50	1.0	2.33 ± 0.90	24
75	0.5	2.23 ± 0.28	25
62.5	1.0	2.06 ± 0.55	26
0	− 0.5	1.90 ± 0.09	27
75	1.0	1.43 ± 0.60	28

Figure 1 was considered as the esthetically most pleasant (ranked as top 1) canine shape by all observers (Table 2 and Fig. 1). The top 2 esthetically most pleasant canine shape collocation was 50% width and 0 mm height; the bottom 1 and bottom 2 esthetically most unpleasant canine shape collocations were 0% width and − 0.5 mm height, and 75% width and 1.0 mm height, respectively (Table 2). The effect of canine edge height (*F* = 486.40, *P* < 0.001) on dental esthetics depended on the canine edge width (*F* = 13.89, *P* < 0.001), and vice versa (*F* = 53.45, *P* < 0.001).

### Subgroup analyses on observers’ background and gender

**Fig. 3:**
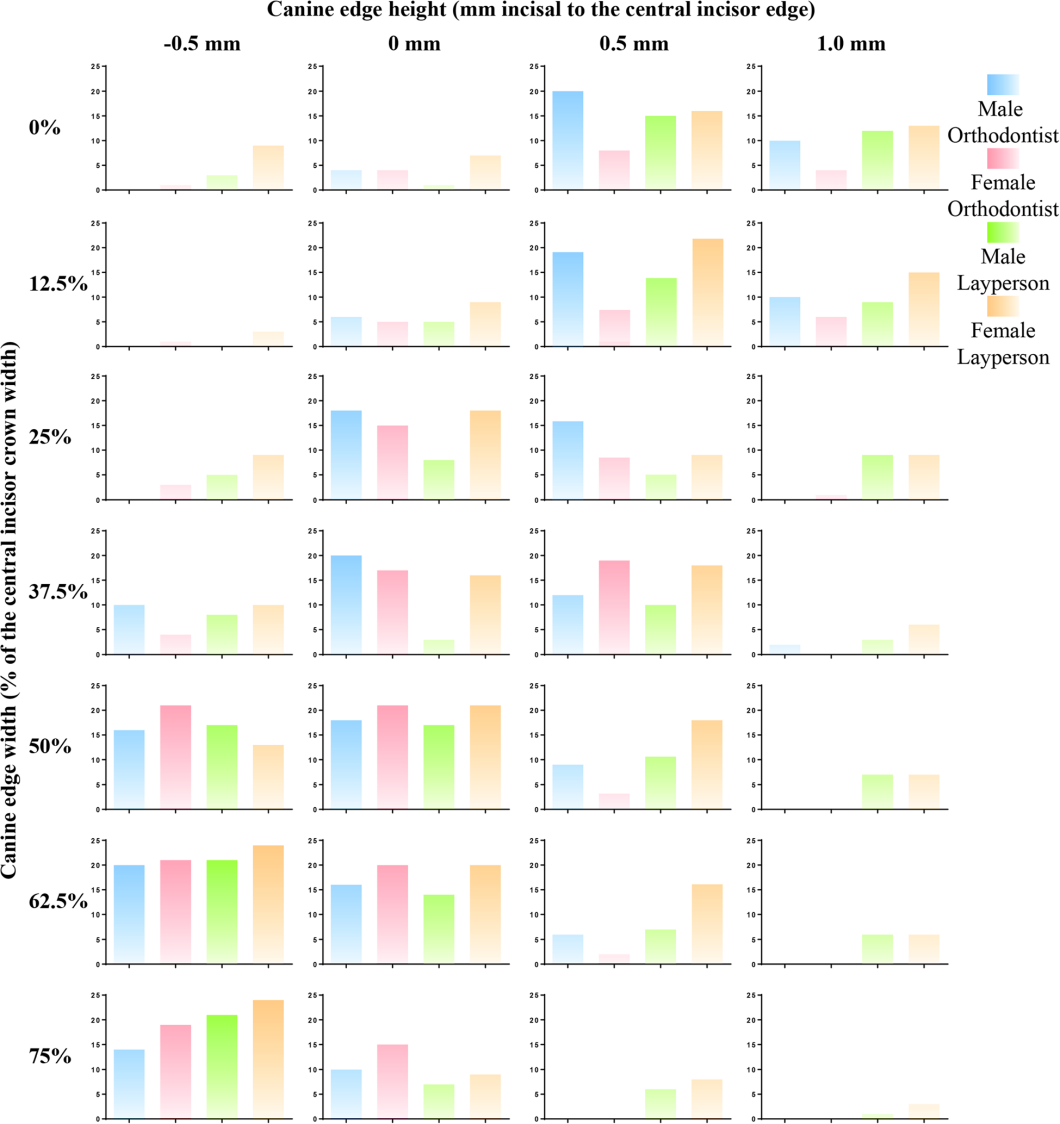
The frequency of the canine shapes that graded as “very attractive” and “attractive” by each subgroup (male orthodontist, female orthodontist, male layperson, and female layperson)

The subgroup analyses on the observers’ background (orthodontist v.s. layperson) and gender (male v.s. female) were performed using the chi-square and Fisher’s exact tests and were summarized in Tables 3 and 4 and Fig. 3.

**Table 3:** Esthetic grade (mean ± standard deviation) and rank on the canine shape by the orthodontists and laypersons. The *P* values were based on the chi-square and Fisher’s exact tests between the orthodontists’ and laypersons’ grades

Canine shape		Orthodontist		Layperson		
Edge width (%)	Edge height (mm)	Grade	Rank	Grade	Rank	*P* values
0	− 0.5	1.67 ± 0.64	27	2.13 ± 1.22	27	0.04
0	0	2.93 ± 0.65	18	2.63 ± 0.87	22	0.59
0	0.5	3.63 ± 0.71	11	3.30 ± 1.16	9	0.02
0	1.0	3.07 ± 0.68	15	3.35 ± 1.05	8	0.55
12.5	− 0.5	2.50 ± 0.60	22	2.35 ± 0.80	26	0.65
12.5	0	3.13 ± 0.58	14	2.70 ± 0.99	20	0.64
12.5	0.5	3.53 ± 0.53	12	3.23 ± 1.07	11	0.42
12.5	1.0	3.40 ± 0.80	13	3.08 ± 1.27	15	0.79
25	−0.5	2.87 ± 0.53	19	2.45 ± 1.08	25	0.06
25	0	3.83 ± 0.60	8	3.45 ± 1.13	5	< 0.01
25	0.5	3.63 ± 0.71	10	2.90 ± 1.17	17	< 0.01
25	1.0	2.53 ± 0.59	21	2.90 ± 1.15	18	< 0.01
37.5	− 0.5	2.97 ± 0.76	17	3.10 ± 1.15	14	0.67
37.5	0	4.23 ± 0.64	5	3.28 ± 1.13	10	< 0.01
37.5	0.5	3.87 ± 0.52	7	2.90 ± 1.08	19	< 0.01
37.5	1.0	2.20 ± 0.49	23	2.68 ± 1.07	21	0.19
50	− 0.5	4.60 ± 0.55	3	3.55 ± 0.99	4	< 0.01
50	0	4.70 ± 0.49	2	3.63 ± 0.87	2	< 0.01
50	0.5	3.03 ± 0.60	16	3.40 ± 0.90	7	0.94
50	1.0	2.00 ± 0.67	25	2.95 ± 1.06	16	< 0.01
62.5	− 0.5	4.80 ± 0.39	1	3.95 ± 0.75	1	< 0.01
62.5	0	4.23 ± 0.58	6	3.57 ± 1.13	3	< 0.01
62.5	0.5	2.70 ± 0.73	20	3.15 ± 1.17	12	0.25
62.5	1.0	1.77 ± 0.42	26	2.45 ± 1.22	24	0.02
75	− 0.5	4.37 ± 0.89	4	3.43 ± 1.13	6	0.16
75	0	3.77 ± 0.82	9	3.13 ± 0.85	13	< 0.01
75	0.5	2.13 ± 0.48	24	2.53 ± 1.15	23	< 0.01
75	1.0	1.00 ± 0.01	28	1.85 ± 1.12	28	0.32

**Table 4:** Esthetic grade (mean ± standard deviation) and rank on the canine shape by the male and female observers. The *P* values were based on the chi-square and Fisher’s exact tests between male and female observers’ grades

Canine shape		Male		Female		
Edge width (%)	Edge height (mm)	Grade	Rank	Grade	Rank	*P* values
0	− 0.5	1.90 ± 0.94	27	2.27 ± 1.12	26	0.32
0	0	2.70 ± 0.86	19	2.88 ± 0.74	19	0.11
0	0.5	3.60 ± 1.18	6	3.33 ± 0.90	12	0.25
0	1.0	3.24 ± 0.96	10	3.22 ± 0.96	15	0.22
12.5	− 0.5	2.30 ± 0.68	26	2.51 ± 0.77	22	0.12
12.5	0	2.78 ± 0.81	18	2.94 ± 0.92	18	0.48
12.5	0.5	3.22 ± 1.02	11	3.49 ± 0.74	8	0.84
12.5	1.0	3.20 ± 1.15	12	3.24 ± 1.12	14	0.16
25	− 0.5	2.38 ± 0.91	23	2.80 ± 0.85	21	0.05
25	0	3.60 ± 1.02	7	3.65 ± 0.93	6	0.02
25	0.5	3.14 ± 1.11	15	3.37 ± 0.85	11	0.54
25	1.0	2.62 ± 0.98	21	2.84 ± 0.97	20	0.99
37.5	− 0.5	3.06 ± 1.08	16	3.04 ± 0.99	16	0.29
37.5	0	3.68 ± 1.09	5	3.48 ± 0.81	9	< 0.01
37.5	0.5	3.16 ± 1.11	13	3.63 ± 0.99	7	0.31
37.5	1.0	2.56 ± 0.92	22	2.43 ± 0.90	23	0.99
50	− 0.5	3.90 ± 0.92	3	3.98 ± 1.02	4	0.99
50	0	3.92 ± 0.93	2	4.24 ± 0.78	2	0.17
50	0.5	3.16 ± 0.76	14	3.31 ± 0.82	13	0.99
50	1.0	2.62 ± 1.04	20	2.31 ± 1.12	25	0.99
62.5	− 0.5	4.10 ± 0.77	1	4.41 ± 0.67	1	0.42
62.5	0	3.54 ± 1.12	8	4.08 ± 0.85	3	0.03
62.5	0.5	2.94 ± 1.09	17	2.98 ± 1.03	17	0.99
62.5	1.0	2.30 ± 0.94	25	2.04 ± 1.06	27	0.99
75	− 0.5	3.74 ± 1.12	4	3.73 ± 1.18	5	0.30
75	0	3.26 ± 0.88	9	3.45 ± 0.91	10	0.16
75	0.5	2.36 ± 0.91	24	2.35 ± 0.97	24	0.99
75	1.0	1.52 ± 0.80	28	1.53 ± 1.01	28	0.62

The orthodontists and laypersons had different esthetic grades and rankings on the canine shapes in general (Table 3 and Fig. 3), with the greatest discrepancy found in the canine shape of 37.5% width and 0.5 mm height (i.e., this canine shape was ranked as the top 7th by the orthodontists but ranked as the 19th by the laypersons). But both orthodontists and laypersons had the same ranking on the top two most esthetic canine shapes (62.5% edge width and − 0.5 mm height, and 50% width and 0 mm height) as well as the bottom two most unaesthetic canine shape (0% width and − 0.5 mm height, and 75% width and 1 mm height).

Male and female observers generally had similar esthetic grades and rankings on the canine shapes (*P* > 0.05). The canine shapes (edge width and height) of 62.5% and − 0.5 mm as well as 50% and 0 mm were also ranked as top 1 and top 2 by both the male and female observers, but their bottom ranking of the most unaesthetic canine shape was the collocations of 75% and 1 mm (Table 4 and Fig. 3).

## Discussion

An esthetic reshapement of the canine plays an important role in a successful canine substitution treatment. This study investigated the influence of the canine edge width and height on dental esthetics and found that the esthetically most pleasant canine edge widths were 50% and 62.5% of the central incisor width and the esthetically most pleasant canine edge heights were 0 mm and − 0.5 mm incisal to the central incisor edge. There was an interaction between the width and height of the canine edge on the dental esthetics, with the 62.5% width and − 0.5 mm height considered to be the esthetically most pleasant canine shape for the canine substitution treatment by all observers in the study.

The shape of maxillary central incisor is generally used as the reference for reshaping the canines during canine substitution treatment [10, 18]. The most esthetic width ratio of the maxillary lateral and central incisors has been considered to vary from 0.62:1 to 0.72:1 [28–31]. The canine edge width of 62.5% of the central incisor width in the study, which was ranked as top 1, was also the closest ratio to the naturally esthetic standard ratio [11, 24].

The canine shapes with relatively pronounced cusps (i.e., 0%–12.5% width and 0.5 mm height; 25%–37.5%width and 0 mm height) were preferred by a considerable number of observers, who regarded them as a symbol of youth and vitality in the study. This is different from the findings of other studies, in which they demonstrated that the esthetic-reshaped canine shares a similar contour with a natural lateral incisor [24, 32] and the deviation may be due to the morphological distinction between the canine and lateral incisor.

The canine edge height has been found to play an important role in the smile arc [33] and gingival margin [34, 35]. The gingival height of canine in our study was relatively more incisal compared with a previous study [34]. It should be noted that the height of gingival margin was digitally altered in the same amount as canine did in the study, but the actual gingival change is usually less than the tooth height change in the real tooth movement due to a delayed or limited periodontal remodeling. Considering that the soft tissues such as gingiva and lips may potentially affect the observers’ grades and rankings on the dental esthetics, the images used in the current study only included the upper dentition [19].

One of the limitations of the study is that the evaluation of dental esthetics was focused on the canine edge (width and height) and was performed on two-dimensional images displaying the upper dentition only. The other canine shape parameters, such as clinical crown width and height, were not assessed in the study. Though we found that the dental esthetics of canine following the canine substitution treatment were influenced by not only the independent parameters of the canine (i.e., edge width and height) but also the different collocations of these two parameters, the other factors, including crown torque [36, 37], enamel color [20, 38, 39], gingival margin [34, 35], and lip position [5], can also affect dental esthetics during the canine substitution treatment. Furthermore, it is important to note that the natural tooth morphology of the anterior teeth can also vary among different ages and genders [40].

Oberservers of different genders and professions may have different expectations in canine substitution treatment cases. In the study, canine shapes of 50–75% edge width and − 0.5–0 mm edge height are widely preferred by all observers. The orthodontists had a greater consistency in grading and ranking the images used in the study than did the laypersons, which was in agreement with the previous studies [20, 41]. This may be because the orthodontists were trained and able to perceive a minor difference in teeth morphology [14, 41]. Additionally, in terms of the pronounced canine cusp (e.g., 0–25% width and 0.5 mm height), the number of male orthodontists showed more preference than did the female orthodontists. In comparison with the orthodontist, the layperson seemed to have less tolerance on the canine shapes with the edge width of 25 to 37.5% and the edge height of 0 mm.

It is also important to note that there were a number of patients and orthodontists who considered a relatively pronounced canine cusp (e.g., 0 to 12.5% width and 0 to 0.5 mm height) to be esthetically attractive. Considering that the canine shape with a relatively pronounced cusp involves relatively less enamel grinding, a customized patient-centered preference of the canine shape (instead of the natural width ratio of the lateral and central incisors) should be applied in practice when treating a patient with canine substitution. Therefore, the involvement of patients in the clinical decisionmaking for the canine reshaping is essential for a successful canine substitution treatment [26, 42].

Another clinical application of the study findings is in the Digital Smile Design (DSD) technology during the Invisalign treatment [43, 44]. A virtual simulation of the tooth movement and shape alteration for patients who need canine substitution would enhance patients’ understanding of different treatment options as well as the communication between patients and clinicians.

Last but not the least, to clinically achieve the most attractive effects, the upper canine replacing the lateral incisor may also receive reshaping with resin, a veneer, or even a crown. Resin restoration can make the canine edge look more similar to the lateral incisor, which however may suffer from discoloration and risk of fall-off. A veneer can reshape not only the edge, but also the labial face of the canine, which is usually more convex compared to a lateral incisor. A crown, with more grinding of the dentin and usually necessitating root canal therapy, can change not only the shape, but also the size of the canine. However, many patients may not choose any of these three approaches, due to the adverse impacts on function (risks when biting hard food with the front teeth) and health (more grinding and even devitalization) of the tooth, and additional costs as well. Therefore, just grinding the canine cusp is an approach of great importance for such cases.

## Conclusions

The most esthetic canine shape for canine substitution is a shape with the edge width of 62.5% of the central incisor crown width and the edge height of 0.5 mm gingival of the central incisor edge. The different collocations of the canine edge width and height affect dental esthetics of the canine during canine substitution treatment. The variation in esthetic preference among different groups of observers highlights the importance of individualized and patient-centered treatment.
